# Whole-Genome Shotgun Sequencing and Characterization of Probiotic Strain Clostridium butyricum UBCB 70 To Assess Its Safety

**DOI:** 10.1128/MRA.01732-18

**Published:** 2019-01-31

**Authors:** Ayesha Sulthana, Archana Thorramamidi, Suvarna G. Lakshmi, Ratna Sudha Madempudi

**Affiliations:** a Center for Research and Development, Unique Biotech Limited, Hyderabad, Telangana, India; Georgia Institute of Technology

## Abstract

Clostridium butyricum is a strictly anaerobic, butyric acid-producing, Gram-positive, spore-forming bacillus that is commonly present in the gut of humans. The complete genome sequence of Clostridium butyricum UBCB 70 was studied to evaluate the presence of antibiotic-resistant and clostridium toxin genes. Here, we announce the draft genome sequence of Clostridium butyricum UBCB 70, isolated from healthy human feces at Unique Biotech Limited, Hyderabad, Telangana, India.

## Abstract (precis)

Clostridium butyricum is a strictly anaerobic, butyric acid-producing, Gram-positive, spore-forming bacillus that is commonly present in the gut of humans. The complete genome sequence of Clostridium butyricum UBCB 70 was studied to evaluate the presence of antibiotic-resistant and clostridium toxin genes.

## ANNOUNCEMENT

A healthy human feces sample was preenriched in modified peptone yeast (PY) medium (1) under anaerobic conditions at 37°C for 5 days. After 3 consecutive dilutions, 0.1 ml of the sample (10**^−^**^3^ dilution) was plated on anaerobic agar (2) and incubated anaerobically at 37°C for 48 h. The isolated pure colony was cultured using reinforced medium (3); the cells were harvested for DNA isolation with the phenol-chloroform extraction method. The 16S rRNA gene was amplified with 27F and 1429R primers (4), followed by amplicon sequencing. Sequence analysis with NCBI BLAST showed 100% sequence similarity with Clostridium butyricum strain TK520 (GenBank accession number CP016332).

Following species identification, the culture pellet of the isolate grown as described above was outsourced for whole-genome shotgun (WGS) sequencing to Chromous Biotech Pvt. Ltd., Bengaluru, Karnataka, India.

The genomic DNA from the culture pellet was fragmented with an ultrasonicator to generate 300 to 400-bp fragments. Then 100 ng of fragmented DNA was used to prepare a paired-end sequencing library with a NEBNext UltraTMII DNA library prep kit, and sequencing was performed on an Illumina HiSeq platform.

A total of 17,652,182 paired-end reads of 150-bp read length on average with a genome coverage of 1,171× were sequenced, out of which 12,864,290 high-quality paired-end reads were filtered with the NGS QC Toolkit (5). The high-quality paired-end reads were assembled into 81 contigs with the *de novo* genome assembler SPAdes version 3.11.1 (6) and the scaffolder SSPACE-standard version 3.0 (7). The draft genome consists of 4,572,221 bp with a GC content of 28.65%, and the largest assembled scaffold is 1,961,597 bp (5). The genome sequence was annotated with the RAST server (8) and the NCBI’s Prokaryotic Genome Annotation Pipeline (PGAP) (9). The genes were predicted and translated through the Prokaryotic Dynamic Programming Gene-finding Algorithm (Prodigal) program (10), following pathway identification with the KEGG Automatic Annotation Server (KAAS) (11). A total of 4,685 genes were predicted, which include 4,177 coding sequences (CDS), 11 rRNAs, and 114 tRNAs. The strain is predicted to encode about 199 proteins involved in carbohydrate metabolism and 150 proteins involved in amino acid metabolism, and 60 genes are involved in the synthesis of proteins and enzymes required for dormancy and sporulation stages. The strain also contains genes involved in the biosynthesis of biotin, riboflavin, cobalamin, thiamine, vitamin B_6_, and folate. The genome was screened to determine the putative virulence factors (virulence factor database [VFDB]) (12), plasmids (PlasmidFinder 2.0) (13), and antibiotic-resistant genes (Antibiotic Resistance Genes Database [ARDB]) (14). The genes encoding putative virulence factors such as botulinum neurotoxin (*atx*), Clostridium difficile toxin (*cdtA*, *cdtB*), hemolytic enterotoxin complex HBL (*hblA*, *hblB*, *hblC*, and *hblD*), nonhemolytic enterotoxin (NHE; *nheA*, *nheB*, and *nheC*), and enterotoxin (*entA*, *entB*, *entC*, and *entD*) were not found. Analysis of the genome sequence of Clostridium butyricum UBCB 70 infers that it does not contain any virulence factors or plasmid-containing antibiotic-resistant genes.

### Data availability.

The raw sequence reads have been submitted to the NCBI SRA under the accession number SRR8372150, and the whole-genome shotgun project of Clostridium butyricum UBCB 70 has been deposited in DDBJ/EMBL/GenBank under the accession number RSEV00000000. The version described in this paper is the first version, RSEV01000000.
